# High levels of pre-treatment HIV drug resistance and treatment failure in Nigerian children

**DOI:** 10.7448/IAS.19.1.21140

**Published:** 2016-11-10

**Authors:** Ragna S Boerma, T Sonia Boender, Kim C.E. Sigaloff, Tobias F Rinke de Wit, Michael Boele van Hensbroek, Nicaise Ndembi, Titilope Adeyemo, Edamisan O Temiye, Akin Osibogun, Pascale Ondoa, Job C Calis, Alani Sulaimon Akanmu

**Affiliations:** 1 Amsterdam Institute for Global Health and Development & Department of Global Health, Academic Medical Centre, University of Amsterdam, Amsterdam, The Netherlands; 2 Global Child Health Group, Emma Children's Hospital, Academic Medical Centre, University of Amsterdam, Amsterdam, The Netherlands; 3 Stichting HIV Monitoring, Amsterdam, The Netherlands; 4 Division of Infectious Diseases, Department of Internal Medicine, Leiden University Medical Centre, Leiden, The Netherlands; 5 Institute of Human Virology Nigeria, Abuja, Nigeria; 6 Lagos University Teaching Hospital, University of Lagos, Lagos, Nigeria; 7 Department of Paediatric Intensive Care, Emma Children's Hospital, Academic Medical Centre, University of Amsterdam, Amsterdam, The Netherlands

**Keywords:** HIV-1, paediatric, HIV drug resistance, PMTCT, sub-Saharan Africa, genotypic resistance testing

## Abstract

**Introduction:**

Pre-treatment HIV drug resistance (PDR) is an increasing problem in sub-Saharan Africa. Children are an especially vulnerable population to develop PDR given that paediatric second-line treatment options are limited. Although monitoring of PDR is important, data on the paediatric prevalence in sub-Saharan Africa and its consequences for treatment outcomes are scarce. We designed a prospective paediatric cohort study to document the prevalence of PDR and its effect on subsequent treatment failure in Nigeria, the country with the second highest number of HIV-infected children in the world.

**Methods:**

HIV-1-infected children ≤12 years, who had not been exposed to drugs for the prevention of mother-to-child transmission (PMTCT), were enrolled between 2012 and 2013, and followed up for 24 months in Lagos, Nigeria. Pre-antiretroviral treatment (ART) population-based *pol* genotypic testing and six-monthly viral load (VL) testing were performed. Logistic regression analysis was used to assess the effect of PDR (World Health Organization (WHO) list for transmitted drug resistance) on subsequent treatment failure (two consecutive VL measurements >1000 cps/ml or death).

**Results:**

Of the total 82 PMTCT-naïve children, 13 (15.9%) had PDR. All 13 children harboured non-nucleoside reverse transcriptase inhibitor (NNRTI) mutations, of whom seven also had nucleoside reverse transcriptase inhibitor resistance. After 24 months, 33% had experienced treatment failure. Treatment failure was associated with PDR and a higher log VL before treatment initiation (adjusted odds ratio (aOR) 7.53 (95%CI 1.61–35.15) and 2.85 (95%CI 1.04–7.78), respectively).

**Discussion:**

PDR was present in one out of six Nigerian children. These high numbers corroborate with recent findings in other African countries. The presence of PDR was relevant as it was the strongest predictor of first-line treatment failure.

**Conclusions:**

Our findings stress the importance of implementing fully active regimens in children living with HIV. This includes the implementation of protease inhibitor (PI)-based first-line ART, as is recommended by the WHO for all HIV-infected children <3 years of age. Overcoming practical barriers to implement PI-based regimens is essential to ensure optimal treatment for HIV-infected children in sub-Saharan Africa. In countries where individual VL or resistance testing is not possible, more attention should be given to paediatric PDR surveys.

## Introduction

Pre-treatment drug resistance (PDR) forms an increasing threat to the success of antiretroviral treatment (ART) programmes in sub-Saharan Africa, where individual resistance testing is not routinely available [1]. In adults, PDR is transmitted mainly through sexual contact with partners previously exposed to antiretroviral drugs (ARVs). Children usually acquire PDR either by direct transmission from the mother or by intra-uterine or perinatal exposure to ARVs as part of prevention of mother-to-child transmission (PMTCT). Given the increasing coverage of PMTCT in sub-Saharan Africa [2], the proportion of HIV-infected children with PDR is likely to grow in the coming years.

As PMTCT usually contains a non-nucleoside reverse transcriptase inhibitor (NNRTI) to which resistance is known to develop quickly [3], the World Health Organization (WHO) previously recommended a protease inhibitor (PI)-based first-line regimen for PMTCT-exposed children. Growing evidence of improved treatment outcomes on PI-based ART for both PMTCT-exposed and unexposed young children [4,5] forced WHO to change their guidelines in 2013, recommending PI-regimens for all children under the age of three years [6]. Many low- and middle-income countries (LMICs) including Nigeria, however, have not yet implemented these recommendations as PI-based regimens are costly and need refrigerated storage, causing logistical challenges. Current national guidelines in Nigeria therefore restrict the use of PIs to children with prior exposure to PMTCT, while all other children start NNRTI-based first-line ART [7].

Determining the PDR rate against NNRTIs and the effect of PDR on treatment outcomes will help answer the question, “to what extent this policy is still defendable?”. Monitoring PDR is especially important in children as they have fewer (second-line) ART options than adults, have higher viral loads (VLs) and will require ART for a longer period. Despite this importance, data on PDR in African children are scarce [8]. This study aimed to document the prevalence of PDR and its effect on treatment outcomes in the first two years of first-line ART in HIV-infected PMTCT-unexposed children in the country with the second highest number of people living with HIV in the world, Nigeria.

## Methods

### Study design and population

The Monitoring Antiretroviral Resistance in Children (MARCH) study is an observational prospective cohort study with a follow-up period of 24 months. HIV-infected children, eligible for ART, and their caregivers were informed about the study and asked to participate at the paediatric HIV clinic and the emergency ward of the Lagos University Teaching Hospital, Nigeria. We aimed to include 90 to 100 children, based on sample size calculations as recommended by the WHO HIVDR survey guidelines [1].

Inclusion criteria were as follows: age ≤12 years, confirmed HIV-1 test (positive HIV antibody test if age >18 months, or a positive HIV nucleic acid polymerase chain reaction (PCR) test if age ≤18 months), eligibility for initiation of first-line ART according to national guidelines at that time (all HIV-infected children <2 years of age, CD4 count <750 cells/m^3^ in children 2 to 5 years and CD4 count <350 cells/mm^3^ in children >5 years) [9] and written informed consent by the parent or guardian. If the child was eight years or older and had disclosed HIV status, assent was required as well. Exclusion criteria were as follows: HIV-2 co-infection, anticipated non-compliance with the protocol and current participation in another study or clinical trial. All children received routine care according to national paediatric HIV treatment guidelines [9]. Clinical and sociodemographic data of mother and child were collected on standardized case report forms at enrolment and every three months thereafter, and laboratory data of the child were collected every six months. All data were source-data-verified by monitors and transferred to a study-specific database. Programmed queries were used to rule out common data errors and inconsistencies. Previous PMTCT exposure was documented as reported in the mother's and/or child's medical files or reported by the child's caregiver. For the current analysis, we included only children who were confirmed to be PMTCT-unexposed in order to exclude any PDR due to previous ARV exposure.

### Laboratory methods

Before ART initiation and every six months during follow-up, a study blood sample (6 ml EDTA tube) was collected for HIV VL testing using the Roche Cobas AmpliPrep TaqMan^®^ (Cobas Amplicor; Roche Diagnostics, Switzerland) to detect virological failure. For all children with a pre-treatment VL >1000 cps/ml, population-based sequencing of the HIV-1 *pol* gene was performed by the reference laboratory of the Institute of Human Virology in Abuja, Nigeria. HIV-1 RNA was extracted from 200 µL plasma, amplified and sequenced as previously described [10] at the National WHO HIV drug resistance reference laboratory of the Institute of Human Virology in Abuja, Nigeria, using an in-house method and primers designed and optimized for CFR02_AG and subtype G isolates [11]. Obtained sequences were visually inspected using Sequencher (Gene Codes Corp., Ann Arbor, MI, USA) at two independent laboratories to verify that each nucleotide base was covered at least by three reads, one of which had to be in the opposite direction of the other two. Sequences were first aligned using HIVAlign (www.hiv.lanl.gov/content/sequence/VIRALIGN/viralign.html). Sequence-genetic relatedness was assessed in MEGA version 5.2.2 (http://www.megasoftware.net/). Samples of which sequences were <1.0% different and had been processed on the same day were re-processed and re-sequenced to rule out cross-sample contamination. To ensure quality of the data set, each sequence was checked before they were submitted to ViroScore^®^ [12]. Major drug resistance mutations were identified based on the 2009 WHO list for surveillance of transmitted resistance [13] using the Stanford Calibrated Population Resistance analysis tool version 6.0 [14]. Susceptibility of the prescribed ART regimen was determined through calculation of the genotypic sensitivity score (GSS) using the Stanford algorithm (Version 7.0) [15]. Reduced susceptibility to the prescribed regimen was defined as GSS<3, that is, <3 fully susceptible drugs. HIV-1 subtyping was performed using the REGA HIV-1 subtyping tool V3 [16]. Genotypic resistance testing was conducted retrospectively and, therefore, results could not be used by the treating physicians to make the basis of their treatment decisions.

The study has received ethical clearance from the Health Research and Ethics Committee of the Lagos University Teaching Hospital, and was conducted in compliance with Good Clinical Practice guidelines and the principles of the Declaration of Helsinki. All laboratory procedures were conducted according to Good Laboratory Practice guidelines.

### Statistical analysis

Patient characteristics were summarized for children with and without PDR separately. Nutritional status was assessed using WHO Anthro (version 3.2.2, January 2011) for children <5 years and WHO Reference 2007 for children ≥5 years [17]. Weight-for-age z-scores and weight-for-height z-scores were only calculated for children <10 years and <5 years of age, respectively. Treatment failure was defined as either two consecutive VL measurements >1000 cps/ml, or death after at least six months of treatment. Children who had a single VL>1000 cps/ml at the last visit of follow-up (and therefore did not have a confirmatory second VL measurement) were considered as having treatment failure as well.

Univariable and multivariable logistic regression was performed to identify children's characteristics associated with PDR and with treatment failure. Explanatory variables considered in the analysis were age, sex, WHO clinical stage, nutritional status, haemoglobin level, CD4 count and CD4 percentage for children ≥5 years and <5 years, respectively, VL, HIV-1 subtype and mother's use of ART. Explanatory variables associated with the outcome variables (*p*<0.20) in the univariable analysis and clinically relevant variables were forwarded to the multivariable model using a step forward procedure. A two-sided *p*-value of ≤0.05 was considered significant. Data were analyzed using Stata 12^®^ (StataCorp LP, TX, USA).

## Results

Between March 2012 and October 2013, 100 children were enrolled in the cohort. Ten children were excluded from the analysis: eight because they were PMTCT-exposed and two because their exposure was unknown. Of the remaining 90 PMTCT-unexposed children, 78 (86.7%) started an ART regimen consisting of zidovudine/lamivudine/nevirapine (AZT+3TC+NVP) as a fixed-dose combination, and none started a PI-based regimen. The median age was 4.6 years (IQR 1.8–8.4), 46% were male and 61% were classified as WHO clinical stage III or IV (Table 1). The child's primary caregiver was the mother for 70 (78%) children. Only four (4.9%) mothers had been diagnosed with HIV before giving birth. The mother was deceased in 15 (17%) cases, including five children (6%) whose both parents had died. The child's median VL before treatment initiation was 160,000 cps/ml (IQR 45,700–730,000); one child had a VL<1000 cps/ml.

**Table 1:** Population characteristics of 90 included children

		Total		No pre-treatment drug resistance		Pre-treatment drug resistance		
					
		*N*=90	%	*N*=69	%	*N*=13	%	*p*
Age	Years (median, IQR)	4.6 (1.8–8.4)		4.5 (1.7–8.7)		4.8 (2.5–6.3)		0.591
	<18 months	19/90	21.1	15/69	21.7	3/13	23.1	0.915
	<3 years	35/90	38.9	28/69	40.6	4/13	30.8	0.508
Sex	Male	41/90	45.6	31/69	44.9	8/13	61.5	0.277
WHO clinical stage	III or IV	55/90	61.1	44/69	63.8	7/13	53.9	0.500
Nutritional status	Stunted, HAZ<−2	21/72	29.2	15/53	28.3	3/12	25.0	0.818
	Wasted, WHZ<−2^a^	12/36	33.3	9/25	36	3/7	42.9	0.741
	Underweight, WAZ<−2^b^	23/78	29.5	19/60	31.7	3/12	25.0	0.648
Haemoglobin	g/dL (mean, SD)	9.8 (1.5)		9.7 (1.3)		10.7 (2.2)		0.055
CD4+ cell percentage^a^	% (median, IQR)	14.9 (8.1–26.1)		16.2 (8.3–26.9)		12.9 (7.9–25.1)		0.505
CD4+ cell count^c^	cells/µL (median, IQR)	393 (137–618)		370 (137–662)		454 (289–587)		0.993
HIV RNA load	log_10_/ml (median, IQR)	5.2 (4.7–5.9)		5.3 (4.8–5.9)		5.0 (4.4–5.6)		0.738
HIV-1 subtype	A	2/82	2.4	2/69	2.9	0/13	0.0	
	C	2/82	2.4	2/69	2.9	0/13	0.0	
	G	31/82	37.8	25/69	36.2	6/13	46.2	
	CRF02_AG	31/82	37.8	28/69	40.6	3/13	23.1	
	Other	16/82	19.5	12/69	17.4	4/13	30.8	0.638
Mother currently on ART	Yes	46/77	59.7	37/64	57.8	6/13	64.3	0.961
ART regimen child	AZT+3TC+EFV	4/90	4.4	3/69	4.4	0/13	0.0	
	AZT+3TC+NVP	78/90	86.7	59/69	85.5	13/13	100	
	ABC+3TC+EFV	1/90	1.1	0/69	0	0/13	0	
	ABC+3TC+NVP	7/90	7.8	7/69	10.1	0/13	0.0	0.759

ART, antiretroviral therapy; HAZ, height for age z-score; IQR, interquartile range; SD, standard deviation; WAZ, weight for age z-score; WHO, World Health Organization; WHZ, weight for height z-score. Genotypic data were available for 82/90 children. Drug resistance mutations were identified based on the 2009 WHO list for surveillance of transmitted drug resistance [12]. HIV-1 subtyping was performed using the REGA HIV-1 subtyping tool V3 [15]. Nutritional status was assessed using WHO Anthro (version 3.2.2, January 2011) for children <5 years and WHO Reference 2007 for children ≥5 years [17]. Results for haemoglobin, CD4 count, CD4 percentage, and HIV RNA load were available for 87, 40, 48, and 82 children, respectively.

a Only for children <5 years of age;

b only for children <10 years of age;

c only for children ≥5 years of age.

**Figure 1:**
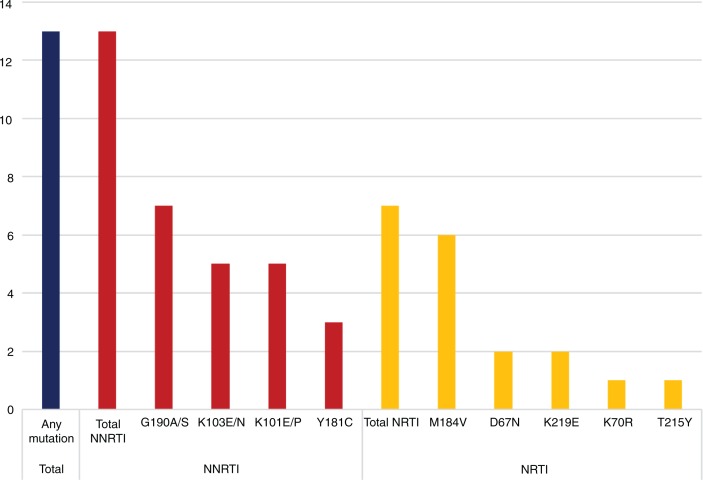
Number of children with pre-treatment drug resistance mutations detected in this cohort (*n=*82). NNRTI: non-nucleoside reverse transcriptase inhibitor; NRTI: nucleoside reverse transcriptase inhibitor.

HIV-1 sequencing of the *pol* gene was successful in 82 children. The children with and without sequencing results did not differ significantly regarding sex, age and clinical characteristics (data not shown). Of 82 children, 13 (15.9%) had PDR; all 13 children carried NNRTI mutations, and seven (8.5%) also had nucleoside reverse transcriptase inhibitor (NRTI) mutations. No PI mutations were identified. G190A/S (*n=*7) and M184V/I (*n=*6) were the most prevalent mutations (Figure 1). For all 13 children with PDR, the virus was predicted to have reduced susceptibility to the treatment prescribed (mean GSS=1.5, SD 0.18). All 13 had mutations associated with high NVP resistance. For all seven children with NRTI mutations, their NRTI backbone was considered less active; five had mutations associated with 3TC resistance and three had thymidine analogue mutations. Children with PDR had a median of two mutations (range 1–7) per sequence. Univariable logistic regression did not detect any significant associations between demographic, clinical or laboratory characteristics and the presence of PDR (Table 1).

After 24 months of follow-up, 25 out of 76 children (32.9%) met the definition of treatment failure; 19 had two consecutive VL measurements >1000 cps/ml and six had a single VL>1000 cps/ml as last measurement; no children died after ≥6 months of treatment. Four children (4.4%) died, all within six months after treatment initiation. For 10 children (11.1%), data on treatment outcomes were incomplete at 24 months: one child transferred out, and nine were lost to follow-up. There was no significant difference in PDR prevalence among those with and without complete follow-up data (data not shown). In children with complete follow-up data, the treatment failure rate was 58.3% (7/12) and 24.6% (14/57) in children with and without PDR, respectively (*p=*0.02). In a multivariable regression analysis, both the presence of PDR (adjusted odds ratio (aOR) 7.53 (95%CI 1.61–35.15), *p=*0.010) and a high VL at treatment initiation (aOR 2.85 (95%CI 1.04–7.78), *p=*0.041 for every log VL increase) were associated with treatment failure (Table 2).

**Table 2:** Factors associated with treatment failure within 24 months of treatment

		Total		Treatment failure		No treatment failure					
								
		*N*=76	%	*N*=25	%	*N=*51	%	Odds ratio	*p*	Adjusted Odds ratio	*p*
Age	Years (median, IQR)	4.6 (1.7–8.3)		2.8 (1.6–6.1)		5.3 (2.0–9.2)		0.88 (0.76–1.01)	0.076	0.92 (0.77–1.09)	0.323
Sex	Male	35/76	46.1	9/25	36.0	26/51	51.0	1.85 (0.69–4.95)	0.221		
WHO clinical stage	III or IV	44/76	57.9	15/25	60.0	29/51	56.9	1.14 (0.43–3.01)	0.795		
Nutritional status	Stunted, HAZ<−2	17/60	29.4	7/19	36.8	10/41	24.4	1.81 (0.56–5.85)	0.322		
	Wasted, WHZ<−2^a^	10/30	33.3	5/13	38.5	5/17	29.4	1.50 (0.33–6.92)	0.603		
	Underweight, WAZ<−2^b^	20/66	30.3	8/22	36.4	12/44	27.3	1.52 (0.51–4.55)	0.450		
CD4+cell percentage^a^	% (median, IQR)	18.8 (10.7–26.4)		15.0 (11.1–30.4)		20.1 (10.7–26.1)		0.98 (0.95–1.01)	0.238		
CD4+cell count^c^	cells/µL (median, IQR)	393 (160–590)		160 (32–587)		405 (236–645)		1.00 (1.00–1.00)	0.342		
HIV RNA load at treatment initiation	log_10_/ml (median, IQR)	5.4 (4.7–5.9)		5.6 (4.9–5.9)		5.2 (4.5–5.9)		1.59 (0.83–3.05)	0.160	2.85 (1.04–7.78)	0.041
Predicted susceptibility to first-line treatment	Reduced	12/69	17.4	7/21	33.3	5/48	10.4	4.30 (1.18–15.72)	0.027	7.53 (1.61–35.15)	0.010

Fourteen children had no available viral load results during follow-up and could not be categorized as failing or not failing treatment. Drug resistance mutations were identified based on the 2009 WHO list for surveillance of transmitted drug resistance [12] and predicted susceptibility to treatment was calculated through the genotypic sensitivity score (GSS). Reduced predicted susceptibility was defined as GSS<3. Nutritional status was assessed using WHO Anthro (version 3.2.2, January 2011) for children <5 years and WHO Reference 2007 for children ≥5 years) [17]. Viral load results, CD4 count and CD4 percentage at treatment initiation were available for 76, 33 and 42 children, respectively. HAZ: Height for age z-score; WAZ: weight for age z-score; WHO: World Health Organization; WHZ: weight for height z-score.

a Only for children <5 years of age;

b only for children <10 years of age;

c only for children ≥5 years of age.

## Discussion

In this cohort of PMTCT-unexposed children in Nigeria, we found a high PDR prevalence of 15.9%. One-third of all children experienced treatment failure in the first two years of first-line ART. Failure occurred in 58% of children with PDR, and multivariable analysis identified PDR as the strongest predictor of subsequent treatment failure increasing the odds of failure 7.5 times.

The high prevalence of PDR we found, seems to fit in a trend of increasing PDR levels in sub-Saharan Africa. Early studies on paediatric PDR in PMTCT-unexposed children show a low prevalence of 0 to 2% [18–20], while more recent studies conducted after 2010 report around 8% [21,22], and a South-African study from 2011 even found that 27 out of 75 unexposed children (36%) had PDR [23]. These paediatric data are in line with the increasing PDR prevalence in adults, correlating with the year of roll-out of national ART programmes and concomitant increased access to ARVs. Among almost 2500 adults in six countries in sub-Saharan Africa, our study group previously reported that the risk of PDR rose by 38% each year after ART roll-out, with the highest prevalence in Uganda, the country with the longest history of large-scale ART provision [24]. A meta-analysis of PDR on a global level (26,102 patients) confirmed these results, and modelled an increase in prevalence of almost 30% per year in East Africa, 14% in southern Africa, and 3% in western and central Africa [25].

The high PDR prevalence we found could be due to the direct transmission of drug-resistant HIV from the mother to her child. The majority of mothers in our cohort were only diagnosed with HIV after giving birth and were ART-naïve; any drug resistance in the mother would therefore be caused by transmitted drug resistance rather than acquired drug resistance. However, it is expected that only women who were recently infected with HIV would be able to transmit a drug-resistant virus, as, without selective pressure of ART, drug-resistant viruses become undetectable soon after infection [26].

Another explanation might be that some of the children who were reported to be PMTCT-unexposed might actually have received PMTCT. However, we attempted to collect all available data on prior PMTCT by interviewing the mother or caregiver and by cross-checking the medical files of all mothers and their children. Furthermore, as most women were not aware of their HIV status during pregnancy, it is unlikely that they received PMTCT. With the increasing coverage of option B (plus) for PMTCT (combination ART for pregnant women) worldwide, the number of perinatally infected children is likely to decrease. However, especially pregnant women with suboptimal adherence to option B (plus) might still transmit HIV to their children, and in those children the chances of PDR are increased. A previous study from South Africa concluded that the PDR rate among children who get perinatally HIV-infected is not expected to decline with the implementation of option B (plus) [23].

Among children with PDR, 58% experienced treatment failure, and multivariable regression analysis identified PDR as the strongest predictor of treatment failure during follow-up. Few other studies have examined treatment outcomes of children with PDR data. In a paediatric cohort in Uganda, in which one-third of children experienced treatment failure in the first 24 months, we also identified PDR and a high pre-treatment VL as independent predictors of treatment failure [22]. Another study in Uganda found PDR in two out of 74 treatment-naïve children. After 48 weeks of treatment, one was virologically suppressed and one was failing treatment, having repeated VL measurements >1000 cps/ml [18]. In adults, a study conducted in six African countries found that PDR was associated with virological failure after 12 months of first-line ART [27].

The second predictor for failure was a high VL before treatment initiation. In children with a very high VL prior to treatment, it will take more time to achieve virological suppression than children with a lower VL [28]. However, we do not expect that this is the mechanism behind the relationship we found, as we defined failure as two consecutive VL>1000 cps/ml after at least six months of ART to allow sufficient time for the VL to decrease in these children. Although the exact mechanism is unclear, the relationship between a high pre-treatment VL and subsequent treatment failure has been described in other paediatric and adult studies [22,27] as well. Physicians should be aware of treatment failure in children with a high VL prior to treatment initiation.

Our study has some limitations. First, data on PMTCT exposure of the included children were collected retrospectively and might be subject to recall bias. Furthermore, children were recruited at a single study site in the capital city of Nigeria, and our data are not necessarily representative for the rest of the country. However, this is only the first paediatric study on PDR in Nigeria, the country with the second highest number of children living with HIV in the world, and our findings urge further research on PDR in other parts of the country. In addition, we cannot exclude the presence of minority variant drug resistance mutations in our cohort, while this has been shown to be associated with an increased risk of virological failure [29,30]. Finally, the children's age ranged from 0 to 12 years, which makes comparison of PDR with other studies harder, as mutation-harbouring variants may have waned to below the assay threshold in older children [23,31]. However, our cohort directly reflects the day-to-day practice of a clinician working in a paediatric HIV clinic, and the practical challenges of HIV drug resistance in a resource-constrained setting.

## Conclusions

Current ART guidelines in Nigeria and many other LMIC still recommend NNRTI-based first-line treatment for PMTCT-unexposed children [4]. The high rate of 16% PDR towards NNRTIs we found in PMTCT-unexposed children implies that one in six children is receiving suboptimal treatment. The majority of children with PDR fail on the prescribed first-line regimen within two years. PDR was the most important predictor of subsequent first-line treatment failure. If this sample is representative of Nigeria, a country with ~260,000 HIV-infected children [32], a PDR prevalence of 16% would have catastrophic implications for paediatric HIV treatment in Nigeria.

These results stress the importance of implementing the WHO recommendations of PI-based regimens for children under three years. As it is known that PDR mutations can be archived in older children [23,31], the actual PDR prevalence in this cohort of children up to 12 years of age is possibly even higher than what we detected. In the near future, we may need to extend the age group that should receive PI regimens to above three years. Paediatric PIs are costlier than NNRTIs and, for young children, it was, until recently, only available as a liquid that requires refrigeration. In 2015, however, the United States Food and Drug Administration (FDA) has approved ritonavir-boosted lopinavir (LPV/r) in pellet form for paediatric usage [33]. This is an important step towards overcoming the barriers of implementing PI-based first-line treatment in resource-constrained settings. Integrase inhibitors, such as raltegravir and dolutegravir, are currently not available for routine paediatric use in LMICs. As soon as access to these drugs in LMICs improves, most notably by decreasing drug prices, integrase inhibitors might be a good alternative to PIs, as these drugs have a high genetic barrier and are associated with less toxicity compared to LPV/r [34].

In many African countries, the detection of treatment failure is difficult as routine VL monitoring is not available. Individual genotypic resistance testing before treatment initiation, as in high-income countries [35], is currently not recommended in resource-constrained settings, mainly due to the high costs and the complexity of testing assays [6]. Ideally, this may change in the future due to efforts to develop simplified tests against lower costs, facilitating individualized testing in resource-limited settings [36]. However, until then, drug resistance surveillance programmes on a national level could provide valuable information on PDR prevalence [37]. This is especially important in children given the high prevalence of paediatric PDR and the relation with treatment failure, and because second-line drugs options are limited in this population. Early detection of increasing levels of PDR in the paediatric population of a country can help policymakers to take informed decisions on national ART guidelines and on the selection of first-line regimens.

In summary, we found a high level of PDR in a cohort of children without prior exposure to PMTCT. PDR represented an important risk factor for treatment failure in children on NNRTI-based first-line ART. Implementation of PI-based regimens for children under three years of age in countries in sub-Saharan Africa is urgently needed and might need to be considered for older children as well. Overcoming the barriers of PI-based treatment in LMICs and close monitoring of PDR through regular surveillance programmes are essential to ensure optimal treatment for HIV-infected children.

## Sequence data

Sequences have been submitted to GenBank and are available under accession numbers KX139312 to KX139401.
